# Anemia and blood transfusion in a surgical intensive care unit

**DOI:** 10.1186/cc9026

**Published:** 2010-05-24

**Authors:** Yasser Sakr, Suzana Lobo, Stefanie Knuepfer, Elizabeth Esser, Michael Bauer, Utz Settmacher, Dagmar Barz, Konrad Reinhart

**Affiliations:** 1 Department of Anesthesiology and Intensive Care, Friedrich Schiller University Hospital, Erlanger Allee 103, Jena, 07743, Germany; 2 Department of General and Vascular Surgery, Friedrich Schiller University Hospital, Erlanger Allee 103, Jena, 07743, Germany; 3 Institution of Transfusion Medicine, Friedrich Schiller University Hospital, Erlanger Allee 103, Jena, 07743, Germany

## Abstract

**Introduction:**

Studies in intensive care unit (ICU) patients have suggested that anemia and blood transfusions can influence outcomes, but these effects have not been widely investigated specifically in surgical ICU patients.

**Methods:**

We retrospectively analyzed the prospectively collected data from all adult patients (>18 years old) admitted to a 50-bed surgical ICU between 1^st ^March 2004 and 30^th ^July 2006.

**Results:**

Of the 5925 patients admitted during the study period, 1833 (30.9%) received a blood transfusion in the ICU. Hemoglobin concentrations were < 9 g/dl on at least one occasion in 57.6% of patients. Lower hemoglobin concentrations were associated with a higher Simplified Acute Physiology Score II and Sequential Organ Failure Assessment score, greater mortality rates, and longer ICU and hospital lengths of stay. Transfused patients had higher ICU (12.5 vs. 3.2%) and hospital (18.3 vs. 6.5%) mortality rates (both p < 0.001) than non-transfused patients. However, ICU and in-hospital mortality rates were similar among transfused and non-transfused matched pairs according to a propensity score (n = 1184 pairs), and after adjustment for possible confounders in a multivariable analysis, higher hemoglobin concentrations (RR 0.97[0.95-0.98], per 1 g/dl, p < 0.001) and blood transfusions (RR 0.96[0.92-0.99], p = 0.031) were independently associated with a lower risk of in-hospital death, especially in patients aged from 66 to 80 years, in patients admitted to the ICU after non-cardiovascular surgery, in patients with higher severity scores, and in patients with severe sepsis.

**Conclusions:**

In this group of surgical ICU patients, anemia was common and was associated with higher morbidity and mortality. Higher hemoglobin concentrations and receipt of a blood transfusion were independently associated with a lower risk of in-hospital death. Randomized control studies are warranted to confirm the potential benefit of blood transfusions in these subpopulations.

## Introduction

Anemia is common in critically ill patients [1-4] and is associated with considerable morbidity and worse outcome [1,3]. Conversely, several studies [1,3] have indicated a potential association between blood transfusion and poor outcome from critical illness. Large observational European [1] and North American [3] cohort studies on blood transfusion practices in critically ill patients reported that blood transfusion was independently associated with an increased risk of death. This association was confirmed in propensity score-matched groups. Studies in trauma patients [5], in patients with burns [6], in patients undergoing cardiac surgery [7], and in patients with acute coronary syndromes [8] have also suggested increased mortality rates associated with blood transfusions.

A landmark study by Hébert and colleagues [9], the transfusion requirements in critically ill patients (TRICC) study, demonstrated that a restrictive strategy of red blood cell (RBC) transfusion was as effective as a liberal strategy. Moreover, these authors [9] reported a survival benefit with the restrictive strategy in patients younger than 55 years and those with acute physiology and chronic health evaluation (APACHE) II scores of 20 or less**. **Similarly, in a recent study in pediatric critically ill patients, Lacroix and colleagues [10] reported that restricting transfusions to patients with a hemoglobin threshold of 7 g/dl was not associated with an increase in adverse events compared with patients transfused according to a trigger of 9.5 g/dl.

Heightened awareness of the possible risks of blood transfusion has led to changes in blood preparation so that blood transfusions may be safer today than they were a decade ago, not only in terms of viral transmission [11,12], but also in terms of transfusion related immunosuppression (TRIM) [12-14]. In particular, leukoreduction, which may reduce some of the negative immunosuppressive effects of transfusions, has been widely implemented [12,15,16]. A recent observational study [2], the sepsis occurrence in acutely ill patients (SOAP) study, showed that in 821 pairs of patients matched according to a propensity score, the 30-day survival rate was higher in the transfusion group than in patients who were not transfused. The effects of blood transfusion need, therefore, to be reassessed following these changes in transfusion preparation and practice.

The aim of our study was to investigate the epidemiology and associated outcome of anemia and blood transfusion in a large cohort of surgical ICU patients.

## Materials and methods

The study was approved by the institutional review board of Friedrich Schiller University Hospital, Jena, Germany. Informed consent was waived due to the retrospective, anonymous nature of the analysis. We retrospectively included all adult (>18 years old) patients admitted to our 50-bed surgical ICU between 1 March 2004 and 30 July 2006. For patients admitted more than once to the ICU only the first admission was considered.

### Data collection

Data were collected from vital sign monitors, ventilators and infusion pumps, and automatically recorded by a clinical information system (Copra System GmbH, Sasbachwalden, Germany). The clinical information system provides staff with complete electronic documentation, order entry (e.g., medications), and direct access to laboratory results. Data recorded prospectively on admission included age, gender, referring facility, primary and secondary admission diagnoses, and surgical procedures. Admission diagnosis was categorized retrospectively on the basis of prospectively recorded codes from the International Classification of Diseases-10 and electronic patient charts.

The simplified acute physiology score (SAPS) II [17] was calculated on admission and the sequential organ failure assessment (SOFA) score [18] calculated daily by the physician in charge of the patient using a special sheet. A plausibility check of the automatically transmitted data was performed by the attending physician before calculating the final scores. In sedated patients, Glasgow Coma Scale prior to initiation of sedation was considered. Hospital mortality and hospital discharge dates were available for all patients from the electronic hospital records.

Blood transfusion was registered electronically in the clinical information system as part of standard procedure in our ICU. Each blood transfusion unit was recorded separately using identification codes that allow tracing in case of suspected or confirmed adverse events. According to our local standards, hemoglobin concentrations should be kept above 7 g/dl in all patients unless blood transfusion is explicitly refused by patients or their next of kin. Hemoglobin concentrations are targeted between 7 to 9 g/dl by administration of one unit of blood at a time followed by determination of hemoglobin concentration. The attending physician may decide to target hemoglobin concentrations above 9 g/dl in the presence of multiple comorbidities, ischemic heart disease, cardiovascular instability, or evidence of tissue hypoperfusion such as increased blood lactate levels or decreased central or mixed venous oxygen saturation. Blood transfusion is discouraged when hemoglobin concentrations are above 10 g/dl. Pre-storage leukodepletion was performed as a standard procedure. Regular quality control checks are performed by the transfusion authorities in our hospital and regular training is given by special personnel.

### Definitions

Comorbidities were defined according to the definitions provided in the original SAPS II paper [17]. SOFAmax was defined as the maximum SOFA score recorded during the ICU stay and SOFAmean as the mean value during the ICU stay [18]. Sepsis syndromes were defined according to consensus conference definitions [19] and their presence was recorded daily by the attending physician in a specific section of the electronic records. Planned admission was defined as an admission after elective surgery that was planned 24 hours before the surgical procedure was conducted.

### Subgroup analysis

*A priori *subgroups were defined arbitrarily according to admission characteristics and included age (18 to 50 years, 51 to 65 years, 66 to 80 years, and more than 80 years), SAPS II score (< 24, 25 to 50, 51 to 75, and more than 75), SOFA score (0 to 4, 5 to 8, 9 to 12, and more than 12), surgical procedures (cardiovascular vs. non-cardiovascular surgery), and the occurrence of severe sepsis.

### Statistical analysis

Data were analyzed using SPSS 13.0 for windows (SPSS Inc, Chicago, IL, USA) and SAS version 9.1.3 software (SAS Institute Inc., Cary, NC, USA). Difference testing between groups was performed using a Wilcoxon test, Mann-Whitney U test, chi-square test and Fisher's exact test as appropriate. A Bonferroni correction was used for multiple comparisons. Analysis of variance was used to assess progression of SOFA score within and among subgroups.

To determine the relative risk of hospital death we developed a multivariable Cox proportional hazard model in the overall population. Variables considered for the Cox regression analysis included age, gender, mechanical ventilation, hemofiltration, referring facility, comorbid diseases, SAPS II and SOFA scores and SOFA subscores on admission, the type of admission (planned or unplanned), the type of surgery, the presence of sepsis during the ICU stay, hemoglobin concentration on admission to the ICU, the minimum hemoglobin concentration during ICU stay, the number of transfused blood units in the ICU, and the maximum number of transfused units within 24 hours during the ICU stay. Colinearity between variables was excluded before modeling. Variables were introduced into this model if significantly associated with a higher risk of in-hospital death on a univariate basis at a *P *less than 0.2 or if clinically relevant variables. To avoid bias related to longer ICU stay in transfused patients, we adjusted for the ICU length of stay (in non-transfused patients) and the time to the first transfusion (in transfused patients). Blood transfusion was introduced in the final model as a time-dependent variable. Another similar Cox regression analysis was performed to evaluate the effects of blood transfusion on in-hospital mortality in subgroups of patients according to gender, age, type of surgery, presence of severe sepsis, and for the different strata of the severity scores.

Propensity scores [20] were obtained through logistic regression of patient characteristics on blood transfusion status, that is, need for blood transfusion as the dependent factor. The propensity score was calculated as the probability based on the final model. A greedy matching technique was used to match individual patients who received a blood transfusion at any time with individual patients who did not, based on propensity scores. The best-matched propensity score was five digits long. Once a match was made, the control patient was removed from the pool. This process was then repeated using four-digit matching, then three-digit matching, and so on. The process proceeded sequentially to a single-digit match on propensity score. If a match was not obtained at this point, the patient who had received a blood transfusion was excluded.

All statistics were two-tailed, and a *P *less than 0.05 was considered to be significant. Continuous variables are presented as mean ± standard deviation or median (25 to 75% interquartile range (IQR)) and categorical variables as number and percentage, unless otherwise indicated.

## Results

A total of 5,925 patients were admitted to our ICU during the study period. The characteristics of the study group are presented in Table 1.

**Table 1:** Characteristics of the study group on admission to the ICU

	All patients
N	5,925
Age, years, mean ± SD	62.2 **± **15.2
Gender, male (%)	3,748 (63.3)
Referring facility	
Operating/recovery room	4,482 (75.7 )
Emergency room	393 (6.6)
Other hospital	30 (0.5)
Other ICU	455 (7.6)
Others	565 (9.6)
Comorbidities (%)	
Diabetes mellitus	1,316 (22.2)
Cancer	1,234 (20.8)
Chronic renal failure	700 (11.9)
COPD	143 (2.4)
Cirrhosis	133 (2.2)
Heart failure (NYHA III to IV)	75 (1.3)
Hematologic cancer	8 (0.1)
Mechanical ventilation (%)	3,248 (54.8)
Severity scores, mean ± SD	
SAPS II score	36.7 **± **18.2
SOFA score	5.9 **± **3.9
Surgery within 24 hours	
Cardiovascular surgery	2,210 (37.3)
General surgery	1,130 (19.1)
Neurosurgery	831 (14.0)
Trauma	342 (5.8 )
Thoracic surgery	260 (4.4)
Others	1,152 (19.4)
Unplanned admissions (%)	1,495 (25.2)
Hemoglobin concentration, g/dl, mean ± SD	9.9 **± **2.3
ICU mortality rate (%)	361 (6.1)
Hospital mortality rate (%)	601 (10.1)
ICU LOS, days, median (IQR)	1 (1-4)
Hospital LOS, days, median (IQR)	12 (9-19)

COPD: chronic obstructive pulmonary disease; IQR: interquartile range; LOS: length of stay; NYHA: New York Heart Association; SAPS: simplified acute physiology score; SD: standard deviation; SOFA: sequential organ failure assessment.

### Hemoglobin concentrations and outcome

**Figure 1:**
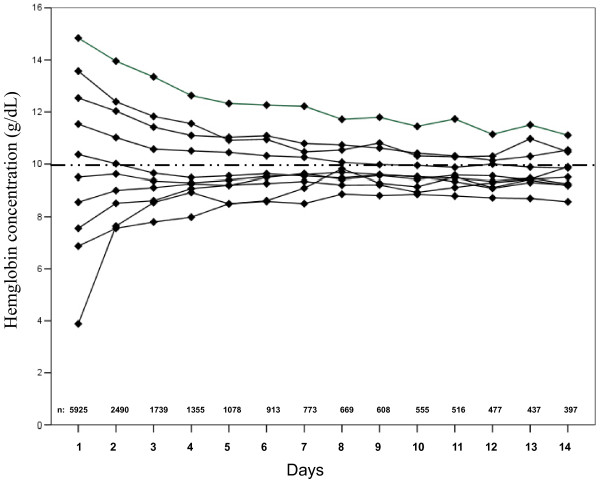
**Time course of hemoglobin concentration during the first two weeks in the ICU**. This was classified according to hemoglobin concentrations on admission (categories with increments of 1 g/dl). Mean values are displayed.

On ICU admission, hemoglobin concentrations were less than 7 g/dl in 18.7% of patients and between 7 and 9 g/dl in 29.5% of patients (mean 9.9 g/dl). During the ICU stay, hemoglobin concentrations were less than 9 g/dl on at least one occasion in 57.6% of patients. Mean hemoglobin concentrations decreased or increased towards median levels of 10 g/dl throughout the first two weeks in the ICU (Figure 1). Patients with hemoglobin concentrations less than 9 g/dl on admission to the ICU had higher SAPS II and SOFA scores than those with higher hemoglobin concentrations [see Table S1 in Additional file 1]. ICU and hospital mortality rates were higher and ICU and hospital lengths of stay were longer in patients with lower hemoglobin concentrations (Table 2). In patients discharged from the ICU (n = 5,564), in-hospital mortality rates were lower in those with higher hemoglobin concentrations on ICU discharge (< 7, 7 to 9, 9.1 to 11, >11 g/dl; 7.3, 7.8, 4.0, and 3.8%, respectively, *P *< 0.001) than those with lower haemoglobin concentrations. SOFA scores increased during the first week in the ICU in all patients [see Figure S1 in Additional file 1]. Patients with hemoglobin concentrations of more than 11 g/dl had the lowest SOFA scores during the first week in the ICU.

**Table 2:** Outcomes according to hemoglobin concentration

	Mortality rates (%)		Length of stay, days median (IQR)	
	ICU	Hospital	ICU LOS	Hospital LOS
Admission hemoglobin concentration	†	†	†	†
< 7 g/dl (n = 1109)	120 (10.8)	171 (15.4)	2 (1-5)	12 (9-21)
7-9 g/dl (n = 1748)	114 (6.5)*	196 (11.2)**	2 (1-4)	13 (10-19)
9-11 g/dl (n = 2021)	85 (4.2)*	158 (6.8)*	1(1-3)*	12 (9-19)
>11 g/dl (n = 1047)	42 (4.0)*	76 (7.3)*	1(1-3)*	11 (7-16)*
Lowest hemoglobin concentration	†	†	†	†
< 7 g/dl (n = 1483)	189 (12.7)	259 (17.5)	3(1-9)	14 (10-25)
7-9 g/dl (n = 1928)	104 (5.4)*	200 (10.4)*	2 (1-5)*	13 (10-20)*
9-11 g/dl (n = 1693)	45 (2.7)*	99 (5.8)*	1 (1-2)*	11 (8-16)*
>11 g/dl (n = 821)	23 (2.8)*	43 (5.2)*	1 (1-1)*	10 (7-14)*

Statistics were performed for columns between categories for initial or minimum hemoglobin concentrations, respectively.

† *P *< 0.001 between groups; * *P *< 0.001 vs < 7 g/dl; ** *P *< 0.05 vs 7 g/dl. IQR, interquartile range; LOS, length of stay.

### Blood transfusion

A total of 1,833 patients (30.9%) received a blood transfusion in the ICU within a median of 1 (IQR 1 to 2) days. The initial blood transfusion was given on the first day in the ICU in 69% of transfused patients (n = 1,209). Transfused patients were older, were more commonly unplanned admissions, had greater SAPS II and SOFA scores, and had a higher incidence of comorbid conditions than patients who were not transfused [see Table S2 in Additional file 1]. The mean hemoglobin concentration prior to transfusion was 8.2 ± 1.4 g/dl (24% at < 7 g/dl, 46.6% at 7 to 9 g/dl, 29.4% at >9 g/dl). Characteristics of patients according to the number of transfused units are presented in Table S3 in Additional file 1.

Transfused patients had higher ICU and in-hospital mortality rates (12.5 vs. 3.2 and 18.3 vs. 6.5%, respectively, both *P *< 0.001 pairwise) and longer ICU and hospital lengths of stay (4 (2 to 11) vs. 1 (1 to 2) and 15 (11 to 26) vs. 11 (8 to 16) days, respectively, both *P *< 0.001 pairwise) than non-transfused patients. There was a relation between the number of transfused units of blood and the degree of organ dysfunction/failure during the ICU stay, as assessed by SOFA scores, length of stay in the ICU, and mortality rates (Table 3). About 50% of patients who received more than eight units of blood died in the hospital. Patients who were transfused later in the ICU stay had higher mortality rates than those who were transfused earlier during the ICU stay (see Figure S2 in the Additional file 1).

**Table 3:** Outcome according to the number of transfused blood units

	1 unit (n = 381)	2 units (n = 683)	3-4 units (n = 378)	5-8 units (n = 224)	>8 units (n = 167)
SOFA scores in the ICU, mean ± SD					
SOFAmax †	7.5 ± 3.3	7.9 ± 3.6*	9.7 ± 3.4*	12.4 ± 3.7*	14.9 ± 3.0*
SOFAmean †	6.3 ± 2.9	6.5 ± 3.2*	7.5 ± 3.1*	8.6 ± 3.6*	9.8 ± 3.3*
ICU LOS, days, median (IQR) ‡	2 (1-5)	3 (1-5)*	4 (2-9)*	13 (6-21)*	28 (15-41)*
Hospital LOS, days, median (IQR)	14 (10-20)	13 (10-22)	15 (11-25)*	20 (13-30)*	34 (20-59)*
Death in ICU (%) †	16 (4.2%)	45 (6.6%)	37 (9.8%)*	58 (25.9%)*	73 (43.7%)*
Death in hospital (%) †	37 (9.7%)	88 (12.9%)	58 (15.3%)*	69 (30.8%)*	84 (50.3%)*

† *P *< 0.001 between groups; ‡ *P *< 0.05 between groups; * *P *< 0.001 vs. patients transfused with one unit of blood. IQR: interquartile range; LOS: length of stay; SD: standard deviation; SOFA: sequential organ failure assessment.

### Multivariable adjustment

In the multivariable Cox regression analysis with in-hospital death as the dependent variable, higher hemoglobin concentrations (relative risk (RR) = 0.97, 95% confidence interval (CI) = 0.95 to 0.98, per 1 g/dl, *P*≤0.001) and receipt of a blood transfusion (RR = 0.96, 95% CI = 0.92 to 0.99, *P *= 0.031) were independently associated with a lower risk of in-hospital death [see Table S4 in Additional file 1].

### Propensity score matching

A total of 1,184 pairs were matched according to their propensity score [see Table S5 and Figure S3 in Additional file 1]. Transfused patients for whom propensity score-matched pairs were found had a higher incidence of chronic renal failure and cirrhosis, were more commonly unplanned admissions, had greater SAPS II and SOFA scores and lower hemoglobin concentrations on admission to the ICU, had higher mortality rates, and longer ICU and hospital lengths of stay than those for whom no matched pairs were found (n = 649) [see Table S6 in Additional file 1]. However, there were no differences in baseline characteristics or outcomes between the propensity score-matched patients (Table 4). The mean hemoglobin concentration prior to transfusion was 8.3 ± 1.8 g/dl in this subgroup. ICU and in-hospital mortality rates were similar (6.3 vs. 7.3% and 11.8 vs. 12.2%, respectively, *P *> 0.2 pairwise) among transfused and non-transfused-matched pairs.

**Table 4:** Basic characteristics and outcome among propensity score matched groups

	Transfusion	No transfusion	*P *value
N	1184	1184	
Age, years, mean ± SD	64.2 **± **15.1	64.9 **±**14.2	0.255
Gender, male (%)	717 (60.6)	709 (59.9)	0.737
Severity scores on admission mean ± SD			
SAPS II score	41.8 **± **16.2	42.2 **± **17.8	0.370
SOFA score	7.1 **± **3.3	7.3 **± **3.5	0.259
Referring facility			0.694
Operating/recovery room	914 (77.2)	886 (74.8)	
Emergency room	55 (4.6)	88 (7.4)	
Other hospital	5 (0.4)	5 (0.4)	
Other ICU	101 (8.5)	88 (7.4)	
Others	109 (9.2)	117 (9.9)	
Comorbidities (%)			
Diabetes mellitus	307 (25.9)	327 (27.6)	0.353
Chronic renal failure	187 (15.8)	178 (15.0)	0.645
Cancer	181 (15.3)	166 (14.0)	0.384
Cirrhosis	28 (2.4)	27 (2.3)	0.892
COPD	31 (2.6)	34 (2.9)	0.706
Heart failure (NYHA III-IV)	18 (1.5)	17 (1.4)	0.865
Hematologic cancer	3 (0.3)	2 (0.2)	0.654
Surgery within 24 hours (%)			0.460
Cardiovascular surgery	563 (47.6)	593 (50.1)	
General surgery	174 (14.7)	133 (11.2)	
Neurosurgery	125 (10.6)	141 (11.9)	
Trauma	94 (7.9)	52 (4.4)	
Thoracic surgery	33 (2.8)	33 (2.8)	
Others	195 (16.5)	232 (19.6)	
Unplanned admissions (%)	357 (30.2)	337 (28.5)	0.367
Hemoglobin concentration on admission to the ICU, mean ± SD	8.4 ± 1.9	8.3 ± 1.7	0.165
Minimum hemoglobin concentration during ICU stay, mean ± SD	8.4 ± 1.9	8.3 ± 1.7	0.219
Severity scores, mean ± SD			
SOFAmean	6.4 **± **3.0	6.5 **± **3.3	0.217
SOFAmax	7.7 **± **3.5	7.5 **± **3.6	0.537
ICU mortality rate (%)	74 (6.3 )	87 (7.3)	0.289
Hospital mortality rate (%)	140 (11.8)	144 (12.2)	0.800
ICU LOS*, median (IQR)	1 (0-3)	1 (1-4)	0.276
Hospital LOS, median (IQR)	12 (9-29)	12 (9-17)	0.201

* ICU LOS in patients who did not receive blood transfusion and the time to the first blood transfusion in transfused patients.

COPD: chronic obstructive pulmonary disease; IQR: interquartile range; LOS: length of stay; NYHA: New York Heart Association; SAPS: simplified acute physiology score; SD: standard deviation; SOFA: sequential organ failure assessment.

### Subgroup analyses

**Figure 2:**
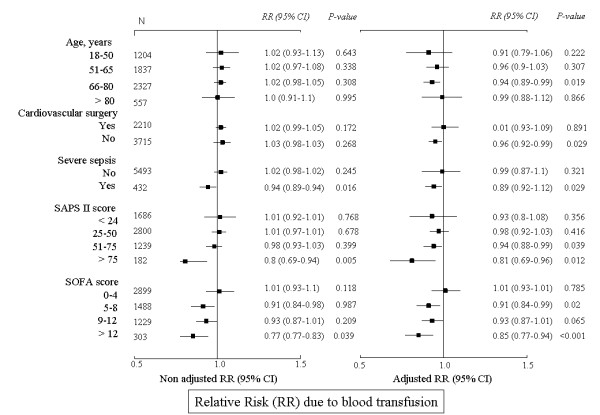
**Relative risk of in-hospital death due to blood transfusion in selected subgroups of ICU patients**. Left panel demonstrates non-adjusted relative risks (RR). Right panel demonstrates relative risks adjusted to age, gender, comorbidities, severity scores on admission to the ICU, referring facility, type of surgery, the presence of sepsis syndromes, hemoglobin concentration on admission to the ICU, and the number of transfused units of blood. Blood transfusion was introduced in the model as a time-dependent variable in relation to the day on which blood transfusion was carried out. CI: confidence interval; SAPS: simplified acute physiology score; SOFA: sequential organ failure assessment.

The results of univariate and multivariable Cox regression analysis in the *a priori *defined subgroups are presented in Figure 2. Blood transfusion was associated with a lower risk of in-hospital death in patients aged from 66 to 80 years, in patients admitted to the ICU after non-cardiovascular surgery, in patients with SAPS II score greater than 50 and SOFA score more than four on admission to the ICU, and in patients with severe sepsis.

## Discussion

In this large cohort of surgical ICU patients, hemoglobin concentrations were less than 9 g/dl on at least one occasion in 57.6% of patients. Lower hemoglobin concentrations were associated with higher morbidity and mortality. In a multivariable analysis, higher hemoglobin concentrations and blood transfusions were independently associated with a lower risk of in-hospital death, especially in patients aged from 66 to 80 years, in patients admitted to the ICU after non-cardiovascular surgery, in patients with higher severity scores, and in patients with severe sepsis.

In this study, we demonstrate that anemia is common in surgical intensive care patients. The cause of anemia in these patients is likely to be multifactorial [4,21]. The retrospective design of our study does not allow us to elaborate on the exact cause of the low hemoglobin concentrations. Nevertheless, we found that lower hemoglobin concentrations were associated with poor outcome even after adjustment for possible confounding factors. Our data confirm the results of previous studies in mixed populations of medical and surgical critically ill patients [1,3], in surgical patients who declined blood transfusions [22,23], and in patients with ischemic heart disease [24,25]. We additionally demonstrate a correlation between hemoglobin concentrations and organ dysfunction/failure as assessed by the SOFA scores in these patients.

Blood transfusion has also been thought to increase the risk of death in ICU patients [1,3]. Indeed, transfused patients in our study had higher ICU and in-hospital mortality rates; however, after adjustment for possible confounders and severity of illness, blood transfusion was associated with a lower risk of in-hospital death. The discrepancy between our results and those of previous observational studies [1,3] may be related to the implementation of leukoreduction in our institution. Hébert and colleagues [15] reported reduced in-hospital mortality rates after implementation of leukoreduction in a large Canadian multicenter study compared with the control period. van de Watering and colleagues [16] showed increased survival rates in post-cardiac surgery patients transfused with packed RBCs filtered to remove leukocytes compared with those transfused with blood just treated to remove buffy coats. Another possible explanation may be the different case-mix in our study from those of the previous observational cohort studies [1,3], which included mixed medical and surgical ICU patients. Nevertheless, our data support those of the recently published analysis from the SOAP study [2], in which blood transfusion, mostly with leukoreduced blood, was associated with a lower RR of death.

In-hospital mortality was the primary end point in our study. This was also the primary end point for previous prospective randomized [15] and observational studies [1,3]. Possible deleterious effects of blood transfusions, especially immunosuppression, are expected to occur later in the course of the disease. The relatively short ICU length of stay in our study may, therefore, render the ICU mortality inadequate in this context.

The results of propensity score matching in our study do not exclude beneficial effects of blood transfusion despite similar outcomes between the matched groups. Severely ill patients were not included in this analysis due to the absence of suitable matched pairs. These patients may be more likely to benefit from blood transfusion, a hypothesis supported by the subgroup analysis in our study. The optimal transfusion trigger in ICU patients has been a matter of controversy. Although randomized controlled trials would be the most appropriate means to investigate this issue, observational studies such as ours can provide insight, generate hypotheses, and complement the results of randomized studies. Randomized controlled studies in which subjects are randomized to two different therapeutic strategies, independent of their needs, are at risk of therapeutic misassignment [26]. Exclusion of subgroups of patients according to study protocol, dropout of others due to declined consent or non-compliance of physicians, and failure of recruitment are all factors that hinder extrapolation of the results of randomized controlled trials to other patient populations with different case mixes. Changes in practice and quality of care over time may be another important factor that necessitates reassessment of current treatment strategies. Although the TRICC study [9] demonstrated that a restrictive strategy of blood transfusion was as effective as a liberal strategy, leukoreduction was not implemented at the time that study was performed. Whether or not the results of the TRICC study have changed transfusion practice in ICUs is unclear. The mean pre-transfusion hemoglobin concentration in our study was 8.2 g/dl, which is similar to a large multicenter observational study [3] performed after the results of the TRICC study were published [9] and the evolution of hemoglobin concentrations in our study was also similar to that reported in this study. This could be explained by the limitations of the TRICC study [9] that may hinder the adoption of the restrictive transfusion strategy in all ICU patients.

We also identified subgroups of patients that are more likely to benefit from blood transfusion, including patients with higher severity of illness and more organ dysfunction. These data may help in guiding transfusion practice in surgical ICU patients, until the results of relevant randomized trials are available.

To the best of our knowledge, our study is the largest to date investigating the impact of anemia and possible risks of blood transfusion in surgical intensive care patients. However, some limitations should be considered. First, our analysis is retrospective in nature and our results are only hypothesis generating. A randomized controlled trial is warranted to clarify this issue. Second, the multivariable analysis does not take into account unmeasured variables and can not establish a cause-effect relation. The confounding effect of unmeasured variables can not be excluded. Nevertheless, many relevant variables were considered. Third, similar to previous observational [1-3] and interventional studies [9,10], the impact of blood transfusions given before and after the ICU stay on outcome was not evaluated and the indication for blood transfusion was not identified. Fourth, the indication for blood transfusion was not considered in our analysis and may have been an important confounding factor. However, indication for blood transfusion is usually influenced by hemoglobin concentrations, comorbidities, and severity of illness, all of which are factors that were considered in our analysis. Finally, the results of our study may not be extrapolated to patients with other case mixes, such as medical patients.

## Conclusions

In this large cohort of surgical intensive care patients, anemia was common and was associated with higher morbidity and mortality. Higher hemoglobin concentrations and blood transfusions were independently associated with a lower risk of in-hospital death, especially in patients aged from 66 to 80 years, in patients admitted to the ICU after non-cardiovascular surgery, in patients with severe sepsis, and in patients with higher SAPS II and SOFA scores on admission to the ICU. Randomized controlled studies are warranted to confirm the potential benefit of blood transfusion in these subpopulations.

## Key messages

• Anemia is common in surgical ICU patients and is associated with higher morbidity and mortality

• Blood transfusions may be potentially beneficial in patients with higher severity scores, in patients aged from 66 to 80 years, in patients admitted to the ICU after non-cardiovascular surgery, and in patients with severe sepsis

• Our data should be regarded as being hypothesis-generating and randomized controlled studies are warranted to reassess transfusion practice in the ICU.

## Abbreviations

APACHE: acute physiology and chronic health evaluation; IQR: interquartile; RBC: red blood cell; RR: relative risk; SAPS: simplified acute physiology score; SOAP: sepsis occurrence in acutely ill patients; SOFA: sequential organ failure assessment; TRICC: transfusion requirements in critically ill patients; TRIM: transfusion-related immunosuppression;

## Competing interests

The authors declare that they have no competing interests.

## Authors' contributions

All authors participated in the design of the study. YS, SL, and SK contributed to data collection. YS analyzed the data. YS and SL drafted the manuscript. EE, MB, US, DB, and KR revised the article. All authors read and approved the final manuscript.

## Supplementary Material

Additional file 1 **Supplementary material**. A word file containing supplementary Tables S1, S2, S3, S4, S5 and S6 and Figures S1, S2 and S3. Click here for file
