# Supplementary material for: Anemia and blood transfusion in a surgical intensive care unit
Source: Crit Care. 2010 May 24;14(3):R92. doi: 10.1186/cc9026 (PMC2911729; doi:10.1186/cc9026)
Supplement: Additional file 1 — Supplementary material. A word file containing supplementary Tables S1, S2, S3, S4, S5 and S6 and Figures S1, S2 and S3. [file cc9026-S1.DOC]

**SUPPLEMENTARY ONLINE MATERIAL**

**Anemia and blood transfusion in a surgical intensive care unit**

Sakr Y, Lobo S, Knuepfer S, Esser E, Bauer M, Settmacher U, Barz D, Reinhart K.

**Online Supplement Table S1.** Baseline characteristics according to hemoglobin levels on admission to the ICU.

|  | < 7 g/dl  n=1109 | 7-9 g/dl  n=1748 | 9-11 g/dl  n=2021 | >11 g/dl  N=1047 |
| --- | --- | --- | --- | --- |
| Age, years, mean ± SD † | 61.8 ±16.4 | 64.3±13.9* | 62.1± 14.8* | 59.3 ±16.2 |
| Sex (Male/Female %) † | 62.7 /37.3 | 61.1/38.9 | 62.5/37.5 | 68.9/31.1** |
| Severity scores on admission mean ± SD |  |  |  |  |
| SAPS II score † | 40.9 ± 19.0 | 40.9 ± 17.8 | 34.3 ± 17.3* | 29.5 ± 16.6* |
| SOFA score † | 7.3 ± 3.9 | 7.1±3.7 | 5.1± 3.6* | 3.7 ± 3.3* |
| Referring facility (%) † |  | * | * | * |
| Operating/recovering room | 833 (75.1) | 1424 (81.5)* | 1565 (77.4) | 660 (63) |
| Emergency room | 70 (6.3) | 78 (4.5) | 107 (5.3) | 138 (13.2) |
| Other hospital | 5 (0.5) | 11 (0.6) | 9 (0.4) | 5 (0.5) |
| Other ICU | 79 (7.1) | 107 (6.1) | 144 (7.1) | 125 (11.9) |
| Others | 122 (11.0) | 128 (7.3) | 196 (9.8) | 119 (11.4) |
| Surgery within 24 h † |  |  | * | * |
| Cardiovascular | 533 (48.1) | 958 (54.8) | 582 (28.8) | 137 (13.1) |
| General surgery | 143 (12.9) | 262 (15) | 495 (24.5) | 230 (22) |
| Trauma | 115 (10.4) | 106 (6.1) | 87 (4.3) | 34 (3.2) |
| Neurosurgery | 78 (7.0) | 145 (8.3) | 363 (18.0) | 245 (23.4) |
| Thoracic surgery | 33 (3.0) | 58 (3.3) | 110 (5.4) | 59 (5.6) |
| Others | 207 (18.7) | 219 (12.5) | 384 (19.0) | 342 (32.7) |
| Comorbidities n (%) ‡ |  |  |  |  |
| 1 | 402 (36.2) | 699 (40.0) | 829 (41.0) | 382 (36.5) |
| 2 | 263 (23.7) | 432 (24.7) | 494 (24.4) | 225 (21.5) |
| > 3 | 82 (7.4) | 154 (8.8) | 163 (8.1) | 58 (5.5) |

SAPS: Simplified Acute Physiology Score; SOFA: Sequential Organ Failure Assessment.

†: p < 0.001 between groups; ‡: p< 0.05 between groups; *: p < 0.001 vs < 7 g/dl; **: p< 0.05 vs 7 g/dl

**Online Supplement Table S2.** Characteristics of the study group on admission to the ICU according to blood transfusion during the ICU stay

|  | **Blood transfusion** | **No blood transfusion** | **p-value** |
| --- | --- | --- | --- |
| N | 1833 | 4092 |  |
| Age, mean ± SD | 64.4 ± 14.9 | 61.2 ± 15.3 | <0.001 |
| Gender, male (%) | 1118 (61.0) | 2630 (64.3) | <0.001 |
| Referring facility |  |  | <0.001 |
| Operating/recovery room | 1363 (74.4) | 3119 (76.2) |  |
| Emergency room | 102 (5.6) | 291 (7.1) |  |
| Other hospital | 13 (0.7) | 17 (0.4) |  |
| Other ICU | 188 (10.2) | 267 (6.6) |  |
| Others | 167 (9.1) | 398 (9.77) |  |
| Comorbidities (%) |  |  |  |
| Diabetes mellitus | 487 (26.6) | 829 (20.3) | <0.001 |
| Chronic renal failure | 362 (19.7) | 336 (8.2) | <0.001 |
| Cancer | 256 (14.0) | 978 (23.9) | <0.001 |
| Cirrhosis | 75 (4.1) | 58 (1.4) | <0.001 |
| COPD | 52 (2.8) | 91 (2.2) | 0.169 |
| Heart failure (NYHA III-IV) | 40 (2.2) | 35 (0.9) | <0.001 |
| Hematologic cancer | 5 (0.3) | 3 (0.1) | 0.117 |
| Mechanical ventilation (%) | 1230 (67.1) | 2018 (49.3) | <0.001 |
| Severity scores, mean ± SD |  |  |  |
| SAPS II score | 46.8 ± 17.8 | 32.1 ± 16.5 | <0.001 |
| SOFA score | 8.4 ± 3.7 | 4.8 ± 3.5 | <0.001 |
| Surgery within 24 hours (%) |  |  | <0.001 |
| Cardiovascular surgery | 871 (47.5) | 1339 (32.7) |  |
| General surgery | 280 (15.3) | 850 (20.8) |  |
| Neurosurgery | 170 (9.3) | 661 (16.2) |  |
| Trauma | 146 (8.0 ) | 196 (4.8) |  |
| Thoracic surgery | 50 (2.7) | 210 (5.1) |  |
| Others | 316 (17.2) | 836 (20.4) |  |
| Unplanned admissions (%) | 647 (35.3) | 848 (20.7) | <0.001 |
| Hemoglobin concentration, mean ± SD | 8.0 ± 1.7 | 10.1 ± 2.0 | <0.001 |
| Do-Not-Resuscitate orders prior to ICU admission (%) | 17 (0.9) | 22 (0.5) | 0.089 |
| End-of-life decisions * (%) |  |  |  |
| On admission | 23 (1.3) | 30 (0.7) | 0.049 |
| At any time during the ICU stay | 153 (8.4) | 98 (1.9) | <0.001 |
| ICU mortality rate (%) | 229 (12.5) | 132 (3.2) | <0.001 |
| Hospital mortality rate (%) | 336 (18.3) | 265 (6.5) | <0.001 |
| ICU LOS, median [IQ] | 4 [2-11] | 1 [1-2] | <0.001 |
| Hospital LOS, median [IQ] | 15 [11-26] | 11 [8-16] | <0.001 |

COPD: Chronic Obstructive Pulmonary Disease; LOS: Length of stay; NYHA: New York Heart Association; SAPS: Simplified Acute Physiology Score; SOFA: Sequential Organ Failure Assessment.

* Including Do-Not-Resuscitate (DNR) orders and decisions to withhold or withdraw life sustaining therapy.

**Online Supplement Table S3.** Basic characteristics according to the number of transfused blood units

|  | **1 unit**  **(n=381)** | **2 units**  **(n=683)** | **3-4 units**  **(n=378)** | **5-8 units**  **(n=224)** | **>8 units**  **(n=167)** |
| --- | --- | --- | --- | --- | --- |
| Age, years, mean ± SD † | 65.8 ±13.9 | 65.2 ±14.7 | 63.8 ±15.0* | 63.2 ±15.6* | 60.8 ±16.3* |
| Sex (Male/Female)† | 56.7/43.3 | 56.2/43.8 | 66.4/33.6** | 67.9/32.1* | 68.8/31.1* |
| Severity scores on admission, mean ± SD | | | | | |
| SAPS II score † | 42.2 ± 16.8 | 43.2 ±17.2** | 48.0 ±16.8* | 54.1 ± 16.3* | 59.1 ± 17.9* |
| SOFA score ‡ | 6.3 ± 2.9 | 6.5 ± 3.15 | 7.5 ± 3.1* | 8.6 ± 3.6* | 9.8 ± 3.3* |
| Referring facility (%) † |  |  | * | * | * |
| Operating/recovering room | 305 (80.0) | 528 (77.3) | 276 (73) | 152 (67.9) | 102 (61.1) |
| Emergency room | 13 (3.4) | 31 (4.5) | 25 (6.6) | 14 (6.25) | 19 (11.4) |
| Other hospital | 2 (0.5) | 3(0.44) | 3(0.8) | 1 (0.45) | 4 (2.4) |
| Other ICU | 35 (9.2) | 45 (6.6) | 44 (11.6) | 33 (14.7) | 31 (18.6) |
| Others | 26 (6.9) | 76 (11.2) | 30 (8.0) | 24 (10.7) | 11 (6.5) |
| Surgery within 24 hours † |  |  |  | * | * |
| Cardiovascular | 195 (51.2) | 348 (50.9) | 179 (47.3) | 92 (41.1) | 57 (34.1) |
| General | 52 (13.6) | 103 (15.1) | 50 (13.3) | 43 (19.2) | 32 (19.2) |
| Neurosurgery | 43(11.3) | 56 (8.2) | 45 (11.9) | 20 (8.9) | 6 (3.6) |
| Trauma | 20 (5.2) | 44 (6.4) | 39 (10.4) | 18 (8.0) | 25 (15.0) |
| Thoracic | 15 (3.9) | 16 (2.34) | 10 (2.6) | 4 (1.9) | 5 (4.3) |
| Others | 56 (14.7) | 116 (17.0) | 55 (14.5) | 47 (21.0) | 42 (25.1) |
| Comorbidities (%) ‡ |  |  |  |  |  |
| 1 | 149 (39.1) | 245 (35.9) | 134 (35.5) | 82 (36.6) | 47 (28.1) |
| 2 | 107 (28.0) | 202 (29.6) | 94 (24.9) | 55 (24.6) | 39 (23.4) |
| > 3 | 36 (9.4) | 67 (9.8) | 33 (8.7) | 22 (9.8) | 18 (10.8) |
| Hemoglobin concentration, g/dl, mean ± SD | 8.9 ±1.7 | 8.5 ± 1.6 | 8.3 ± 1.8 | 8.2 ± 1.9 | 8.4 ± 1.9 |

SAPS: Simplified Acute Physiology Score; SOFA: Sequential Organ Failure Assessment.

†: p < 0.001 between groups; ‡: p< 0.05 between groups; *: p < 0.001 vs. patients transfused with 1 unit of blood; **: p< 0.05 vs. patients transfused with 1 unit of blood.

**Online Supplement Table S4.** Cox proportional hazard analysis with time to in-hospital mortality as the dependent variable

|  | Univariate | | Multivariable | |
| --- | --- | --- | --- | --- |
| Variable | RR (95% CI) | p-value | RR (95% CI) | p-value |
| Age (per year) | 1.03 (1.02-1.04) | <0.001 | 1.34 (1.21-1.49) | <0.001 |
| Gender (Female) | 1.17 (0.99-1.38) | 0.055 | 1.07 (0.89-1.27) | 0.446 |
| Unplanned admissions | 1.66 (1.40-1.95) | <0.001 | 0.88 (0.72-1.07) | 0.230 |
| Comorbidities |  |  |  |  |
| Cancer | 1.08 (0.89-1.31) | 0.393 | 1.21 (0.94-1.57) | 0.135 |
| Hematological cancer | 0.49 (0.06-3.56) | 0.486 | 1.03 (0.14-7.40) | 0.976 |
| Heart failure (NYHA III-IV) | 1.77 (1.02-3.07) | 0.041 | 1.01 (0.54-1.89) | 0.964 |
| Cirrhosis | 2.25 (1.60-3.17) | <0.001 | 1.67 (1.16- 2.42) | 0.006 |
| Chronic renal failure | 2.13 (1.75-2.59) | <0.001 | 1.41 (1.14-1.74) | 0.002 |
| Diabetes mellitus | 1.41 (1.18-1.68) | <0.001 | 1.17 (0.96-1.42) | 0.106 |
| Referring facility |  |  |  |  |
| OR/recovery | Reference | - | Reference | - |
| Emergency room | 1.78 (1.36-2.33) | <0.001 | 1.50 (1.09-2.06) | 0.011 |
| Other hospital | 2.30 (1.09-4.88) | 0.029 | 1.29 (0.59-2.79) | 0.515 |
| Other ICU | 2.17 (1.72-2.73) | <0.001 | 1.25 (0.93-1.69) | 0.135 |
| Surgery within 24 hours |  |  |  |  |
| Cardiovascular | Reference | - | Reference | - |
| Neurosurgery | 0.83 (0.62-1.13) | 0.256 | 1.61 (1.14-2.27) | 0.006 |
| Thoracic surgery | 0.98 (0.60-1.58) | 0.941 | 1.30 (0.76-2.23) | 0.328 |
| General surgery | 1.45 (1.14-1.83) | 0.002 | 2.27 (1.70-3.04) | <0.001 |
| Trauma | 0.93 (0.63-1.35) | 0.932 | 1.31 (0.86-1.97) | 0.199 |
| Mechanical ventilation | 1.35 (1.14-1.59) | <0.001 | 0.98 (0.81-1.19) | 0.853 |
| Hemofiltration | 4.74 (3.69-6.11) | <0.001 | 2.10 (1.57-2.82) | <0.001 |
| Sepsis syndromes in the ICU |  |  |  |  |
| No sepsis | Reference | - | Reference | - |
| SIRS | 1.31 (1.10-1.57) | 0.002 | 1.10 (0.91-1.33) | 0.296 |
| Sepsis | 2.06 (1.38-3.05) | <0.001 | 1.12 (0.73-1.70) | 0.594 |
| Severe sepsis | 2.40 (1.12-5.11) | 0.023 | 0.78 (0.34-1.80) | 0.573 |
| Septic shock | 3.17 (2.25-4.45) | <0.001 | 0.85 (0.58-1.25) | 0.430 |
| ICU LOS (days)** | 1.60 (1.28-2.00) | <0.001 | 1.34 (1.21-1.49) | <0.001 |
| Hemoglobin concentration on admission to the ICU ¶ | 0.96 (0.95-0.97) | <0.001 | 0.97 (0.95-0.98) | <0.001 |
| Nadir hemoglobin concentration during ICU stay ¶ | 0.94 (0.95-0.97) | <0.001 | 0.96 (0.95-0.98) | <0.001 |
| SAPS II score (per 10 points) †‡ | 1.05 (1.05-1.05) | <0.001 | 1.05 (1.04-1.05) | <0.001 |
| SOFA score (per point) †‡ | 1.02 (0.01–1.04) | <0.001 | 1.02 (0.01–1.05) | <0.001 |
| SOFA subscores (per point) †‡ |  |  |  |  |
| Respiratory | 1.14 (1.09-1.21) | <0.001 | 1.01 (0.94-1.08) | 0.898 |
| Coagulation | 1.37 (1.25-1.49) | <0.001 | 1.19 (1.06-1.34) | 0.003 |
| Hepatic | 1.38 (1.27-1.50) | <0.001 | 1.17 (1.05-1.32) | 0.007 |
| Cardiovascular | 1.22 (1.16-1.28) | <0.001 | 1.22 (1.12-1.34) | <0.001 |
| Neurological | 1.18 (1.12-1.23) | <0.001 | 1.18 (1.11-1.26) | <0.001 |
| Renal | 1.49 (1.38-1.59) | <0.001 | 1.35 (1.23-1.49) | <0.001 |
| Number of transfused units § | 1.21 (1.05-2.23) | 0.004 | 1.25 (0.73-1.58) | 0.462 |
| Max number of transfused units within 24 hours § | 1.17 (1.01-1.85) | <0.001 | 1.01 (0.76-1.32) | 0.529 |
| Blood transfusion* | 1.02 (0.99-1.05) | 0.069 | 0.96 (0.92-0.99) | 0.031 |

LOS: Length of stay; NYHA: New York Heart Association; SAPS: Simplified Acute Physiology Score; SIRS: Systemic Inflammatory Response Syndrome.

* Introduced in the model as a time dependent variable in relation to the day in which the first blood transfusion was carried out.

** ICU length of stay in non transfused patients and the day to the first transfusion in transfused patients.

†, ¶, § Introduced alternatively in the model due to colinearity (R>0.7)

‡ Replacing values in transfused patients with SAPS II or SOFA scores at the time of first transfusions does not alter the significance of any covariate

**Online Supplement Table S5.** Logistic regression model used to generate propensity score with blood transfusion as the dependent variable.*

|  | Coefficient | SEM | Wald | Odds ratio (95% CI) | p-value |
| --- | --- | --- | --- | --- | --- |
| Age (per year) | 0.010 | 0.003 | 10.7 | 1.01 (1.01 - 1.02) | 0.001 |
| Hemoglobin concentration (g/dl’) | -0.180 | 0.007 | 669.9 | 0.84 (0.82 – 0.85)- | <0.001 |
| Unplanned admission | 0.146 | 0.093 | 2.5 | 1.16 ( 0.97 – 1.39) | 0.115 |
| Gender (female) | 0.269 | 0.077 | 12.2 | 1.31 (1.13 – 1.52) | <0.001 |
| Cardiovascular surgery | -0.371 | 0.092 | 16.4 | 0.69 (0.58 – 0.83) | <0.001 |
| Referring facility |  |  |  |  |  |
| Operating room | Reference | - | - | - | - |
| Emergency room | -0.913 | 0.191 | 22.9 | 0.4 (0.28 – 0.58) | <0.001 |
| Other hospital | -0.229 | 0.533 | 0.9 | 0.8 (0.28 – 2.26) | 0.667 |
| Other ICU | 0.060 | 0.150 | 0.2 | 1.06 (0.79 – 1.42) | 0.691 |
| Others | -0.166 | 0.134 | 1.5 | 0.85 (0.65 – 1.1) | 0.217 |
| Cancer | 0.277 | 0.109 | 6.4 | 1.32 (1.61 – 1.63) | 0.011 |
| Hematological cancer | 2.207 | 0.875 | 6.4 | 9.09 (1.63 – 50.5) | 0.012 |
| Heart failure (NYHA III-IV) | 0.362 | 0.302 | 1.4 | 1.44 (0.79 – 2.59) | 0.232 |
| Cirrhosis | 0.794 | 0.235 | 11.4 | 2.21 (1.4 – 3.51) | 0.001 |
| Chronic renal failure | 0.277 | 0.111 | 6.2 | 1.32 (1.06 – 1.64) | 0.013 |
| Diabetes mellitus | 0.188 | 0.087 | 4.7 | 1.21 (1.02- 1.43) | 0.030 |
| SAPS II score (per point) | 0.011 | 0.003 | 9.2 | 1.01 (1.01 – 1.02) | 0.002 |
| SOFA score (per point) | 0.112 | 0.017 | 45.3 | 1.12 (1.08 – 1.16) | <0.001 |
| ICU LOS † | 0.136 | 0.009 | 230.3 | 1.15 (1.13 – 1.17) | <0.001 |
| Severe sepsis | 0.887 | 0.179 | 24.7 | 2.43 (1.71 – 3.45) | <0.001 |
| Constant | 4.237 | 0.555 | 58.2 | NA | NA |

LOS: Length of stay; SAPS: Simplified Acute Physiology Score; SOFA: Sequential Organ Failure Assessment.

CI: confidence interval, NYHA: New York Heart Association criteria, SEM: standard error of mean.

* Hosmer and Lemshow goodness of fit Chi square 12.5 (p=0.09), Nagelkerke pseudo R^2^=0.5. Area under receiver operating curve (AUC): 0.88.

† ICU LOS in patients who did not receive blood transfusion and the day in which patients received the first unit of blood transfusion in the transfused group.

**Online Supplement Table S6.** Baseline characteristics and outcome in transfused patients for whom a propensity score matched pair could be identified (n=1184) and those who did not have a propensity score matched pair.

|  | **Matched pair found** | **No matched pair found** | **p-value** |
| --- | --- | --- | --- |
| N | 1184 | 649 |  |
| Age, mean ± SD | 64.2 ± 15.1 | 64.8 ±14.6 | 0.263 |
| Gender, male (%) | 717 (60.6) | 401 (61.8%) | 0.606 |
| Referring facility |  |  | 0.009 |
| Operating/recovery room | 914 (77.2) | 449 (69.2) |  |
| Emergency room | 55 (4.6) | 47 (7.2) |  |
| Other hospital | 5 (0.4) | 8 (1.2) |  |
| Other ICU | 101 (8.5) | 87(13.4) |  |
| Others | 109 (9.2) | 58(8.9) |  |
| Comorbidities (%) |  |  |  |
| Diabetes mellitus | 307 (25.9) | 180 (27.7) | 0.403 |
| Chronic renal failure | 187 (15.8) | 175 (27.0) | <0.001 |
| Cancer | 181 (15.3) | 75 (11.6) | 0.029 |
| Cirrhosis | 28 (2.4) | 47 (7.2) | <0.001 |
| COPD | 31 (2.6) | 21 (3.2) | 0.446 |
| Heart failure (NYHA III-IV) | 18 (1.5) | 22 (3.4) | 0.009 |
| Mechanical ventilation (%) | 771 (65.1) | 459 (70.7) | 0.015 |
| Severity scores, mean ± SD |  |  |  |
| SAPS II score | 41.8 + 16.2 | 55.9 + 16.9 | <0.001 |
| SOFA score | 6.4 + 3.02 | 8.8 + 3.4 | <0.001 |
| Surgery within 24 hours (%) |  |  | 0.009 |
| Cardiovascular surgery | 563 (47.6) | 308 (47.5) |  |
| General surgery | 174 (14.7) | 106 (16.3) |  |
| Neurosurgery | 125 (10.6) | 845 (6.9) |  |
| Trauma | 94 (7.9) | 52 (8.0) |  |
| Thoracic surgery | 33 (2.8) | 17 (2.6) |  |
| Others | 195 (16.5) | 121 (18.6) |  |
| Unplanned admissions (%) | 357 (30.2) | 290 (44.7) | <0.001 |
| Hemoglobin concentration, g/dl, mean ± SD | 11.1 + 2.1 | 10.2 + 2.2 | <0.001 |
| ICU mortality rate (%) | 74 (6.3) | 155 (23.9) | <0.001 |
| Hospital mortality rate (%) | 140 (11.8) | 196 (30.2) | <0.001 |
| ICU LOS, median [IQ] | 2 [1-5] | 14 [10-21] | <0.001 |
| Hospital LOS, median [IQ] | 12[4-25] | 21[12-36] | <0.001 |

IQ: interquartile range; COPD: Chronic Obstructive Pulmonary Disease; LOS: Length of stay; NYHA: New York Heart Association; SAPS: Simplified Acute Physiology Score; SOFA: Sequential Organ Failure Assessment.

**Online Supplement Figure S1**: Time course of SOFA score during the first week in the ICU according to hemoglobin concentration on admission to the ICU; circles < 7 g/dl; triangles 7-9 g/dl; squares 9-11 g/dl; diamonds > 11 g/dl. Multifactorial analysis of variance (ANOVA) p=0.002 overtime between groups. * ANOVA: p <0.01 within group over time; † ANOVA: p <0.01 compared to patients with hemoglobin concentration > 11 g/dl; ‡ ANOVA: p <0.01 compared to patients with hemoglobin concentration 9-11 g/dl.

**Online Supplement Figure S2**: Bar chart representing ICU and hospital mortality rates (%) according to the time of the first transfusion in the ICU. ICU mortality; * p <0.05 compared to the first category (Chi square test with Bonferroni correction for multiple comparisons). Hospital mortality; † p <0.05 compared to the first category (Chi square test with Bonferroni correction for multiple comparisons).

The transfusion trigger ranged between 8.16 to 8.31 g/dL between categories (p=361).

**Online Supplement Figure S3**. Receiver operating curve demonstrating the discrimination of propensity score on blood transfusion. Area under the curve (AUC) = 0.88
